# Notches on the dial: a call to action to develop plain language communication with the public about users and uses of health data

**DOI:** 10.23889/ijpds.v4i1.1106

**Published:** 2019-08-05

**Authors:** PA Paprica, KM McGrail, MJ Schull

**Affiliations:** 1 Institute of Health Policy, Management and Evaluation, University of Toronto, Health Sciences Building, 155 College Street, Suite 425, Toronto, ON M5T 3M6; 2 University of British Columbia, UBC Centre for Health Services and Policy Research Vancouver Campus 201-2206 East Mall Vancouver, BC Canada V6T 1Z3; 3 Institute for Clinical Evaluative Sciences (ICES), G1 06, 2075 Bayview Avenue, Toronto, Ontario M4N 3M5

Population data science [1] researchers are not alone in recognizing the value of health and health-related data. In the era of big data, and with advent of machine learning and other artificial intelligence methods, organizations around the world are actively working to turn data into knowledge, and, in some cases, profit. The media and members of the public have taken notice, with high profile news stories about data breaches and privacy concerns [2—4] alongside some stories that call for increased use of data [5,6]. In response, public and private sector data-holding organizations and jurisdictions are turning their attention to policies, processes and regulations intended to ensure that personal data are used in ways that that the public supports. In some cases, these efforts include involving “publics” in decisions about data, such as using patient and lay person advice and other inputs to help shape policies [7-10].

Public-facing communications about data, including those related to Europe’s General Data Protection Act (GDPR) [11] and Canada’s Digital Charter [12], emphasize individual-level consent and the responsibility of businesses to protect privacy. However, not all data require individual consent for secondary use, and businesses are not the only users of person-level data. For example, in Canada, public-sector privacy legislation tends to include allowances for research and statistical uses of data without consent under specific circumstances [13,14]. Under the GDPR, consent is just one of the lawful bases that can be relied upon for data processing, and there are provisions for tasks carried out in the public interest, which can include scientific research [15,16].

It is our view that there has been insufficient communication with the public about data-intensive health research, particularly that performed by public sector researchers using non-consented health and health-related data. The stakes are high. Consented data will always be a subset of all the data, with important differences in terms of age, sex, race, income, education, and/or health status between people who consent to participate in health research studies and those who do not [17,18]. As data use and data literacy increases, if the research community is seen as failing to adequately inform the public about the fact that non-consented data are used in studies, we risk losing public trust, support, and funding. The result could be extreme negative consequences, potentially shutting off many avenues of population research, including research related to vulnerable sub-populations who are underrepresented in consented datasets.

We believe that achieving effective proportionate governance of health data [19] requires authentic public and patient involvement that follows accepted principles such as inclusiveness, two-way communication and transparency [20]. There is a growing body of research evidence about public expectations around social licence and acceptable data uses of health data [21—24], and increasing commitment from many institutions to include the public in one way or another to inform or influence policies [7—10], but we have not yet implemented or operationalized the principles and ideas presented in the research literature at scale. One approach, presented here, is to create some standardized communications that distinguish between different uses of health data to ensure that members of the public do not confuse, or group together, commercial revenue-generating uses with public sector data-intensive health research. For example, we might aim to distinguish between different uses of health data such as:

- An organization using data generated through the services that they deliver, without consent, to inform decisions about their core business (e.g., an insurance company using client data to develop new products or investigate potential fraud, or a hospital using the data it generates to improve the quality of its services)
- An organization providing the data that they generate or collect to another organization, with consent of the data subject, in exchange for money or some other anticipated financial benefit (e.g., a company that provides genetic testing services to the general public selling client data to a pharmaceutical company where the company can demonstrate that they have clients’ consent for the transaction)
- Private or public sector led research studies under which all participants have provided consent for their data to be used for a particular purpose (e.g., a clinical trial of a new drug product led by a pharmaceutical company)
- Private or public sector led research studies that make use of data infrastructure established with participants’ consent for multiple uses including, in some cases, unspecified future uses (e.g., an academic study of interactions between genomes and the environment that uses data from the Canadian Partnership for Tomorrow Project or the UK Biobank)
- Private or public sector led research studies that use non-consented data from population-wide datasets in order to get a complete picture of a health issue (e.g., a study of an epidemic led by an academic researcher that uses non-consented data without identifiers for the entire population)

Existing research evidence can help us develop plain language communication focused on the factors that the public cares the most about. The international research literature describes general but conditional public support for data-intensive health research. Qualitative studies indicate that members of the public view health data as an asset that should be used as long as there is a public benefit and their concerns related to privacy, commercial motives and other risks are addressed [21—24]. The Wellcome Trust, Ipsos Mori One-Way Mirror Report identifies four ‘key tests’ for public acceptability of commercial use of health data [24]:

- WHY – Is it for a particular public benefit and not just private profit?
- WHO – Can the people using my data be trusted to produce a public benefit?
- WHAT – Am I giving sensitive data? Could it be linked back to me?
- HOW – Are there safeguards in place to keep my data private and secure?

There is also guidance for governance and management of access to sensitive data, with the Five Safes framework emerging as an international standard [25]. The Five Safes framework is currently in use in the UK, Australia, New Zealand and Canada, and likely beyond [25—28]. The framework is not prescriptive, and provides a broad way to think about what would be acceptable and appropriate use of data. The components of the Five Safes framework are similar in content to those in the One-Way Mirror report with increased emphasis on factors that the scientific community sees as important, and include:

- Safe Projects – Is there scientific merit? Is there public value?
- Safe People – Who is using the data? What training do they have?
- Safe Data – How potentially identifiable are the data? Is there consent? Is there legal authority for use?
- Safe Settings – Where will be the data be analyzed? How will they be managed?
- Safe Outputs – Is there any potential disclosure, either of individuals, families or communities?

Notwithstanding this foundation of research, most of the information about data that is currently available to the public comes in the form of very long terms of use and privacy policies, such as Google’s 27-page privacy policy [29], which technically provide all the information that is legally required, but practically speaking, are very hard for members of the public to process or understand [30,31].

Synthesizing information from the references presented in this Commentary, it seems likely that we could do much better by focusing communication about health data on basic facts such as:

1. Whether and how people can opt-out of having their data used. This would include plain language information about how they can exercise that right where it exists, and why there are cases where it does not.
2. A statement about whether the data that people contribute will be used to generate revenue which also distinguishes between revenue generated for profit and cost-recovery charges.
3. Text that makes it clear who will have access to or “touch” the data, e.g.:
  - Only individuals within the organization that is collecting/receiving the data
  - Public sector (academic) researchers with Research Ethics Board approval (or equivalent)
  - Private sector researchers with Research Ethics Board approval (or equivalent)
  - Third party organizations and individuals who want to use the data, including for market research and non-research purposes
4. Information about the main privacy and security safeguards that are in place, including where any analysis of the data will take place and what training users of data receive.
5. A statement about what sort of data are being used and how personally identifiable they are.
6. A plain language statement about why the data are being used, e.g., the public or societal benefits that may be realized or the benefits to the company that is working with the data.
7. Where it is not otherwise obvious, a statement about whether the organization that has created the communication is a commercial for-profit-entity, a government department or agency, an academic institution, a not-for-profit corporation or some combination of organizations.

This list is not meant to be exhaustive or definitive, and there may be additional categories and better ways to present it. For example, imagine how transparency would be improved if a fictional commercial organization “ABC” which earns revenue from the sale of client data provided the text in Box 1 as a complement to its (longer) privacy policy or terms of use agreement:

Box 1: Draft example of plain language communication about fictional commercial organization “ABC” which earns revenue from data and provides data to third parties At ABC we use your data to improve our products and services. [ADDRESSING: WHY; SAFE PROJECTS]

Less than 100 of ABC’s 3,000 staff have access to identifying information such as your name and address; other staff at ABC work with pseudo-anonymized datasets that don’t include names or other identifying information. [ADDRESSING: WHO; SAFE PEOPLE/DATA]

We earn 5-10% of our annual revenue from the data we hold. In some cases, we provide identified data to other companies which includes your name and contact information, most of the time we perform analytic services for other companies and provide them with summary statistics. We invest approximately half of the revenue we earn from data in maintaining our databases and ensuring the privacy and security of data holdings. [ADDRESSING: WHAT/HOW; SAFE DATA/SETTINGS/OUTPUTS]

Academic researchers with Research Ethics Board Approval and start-up companies under the government’s YYY program also have access to pseudo-anonymized data that doesn’t include identifying information which are held in a data trust managed by ZZZ. [ADDRESSING: WHO/WHAT/HOW; SAFE PROJECTS/PEOPLE/DATA/SETTINGS]

For information about which uses of data that you can opt out of, and how to opt out, click **here**.

Our full privacy policy is available **here**.

Interestingly, with the exception of the text about providing identified data to third parties, the text for a public sector research organization that provides access to data could be quite similar to the text in Box 1, noting the first purpose statement or benefit of using data would likely be knowledge generation or research studies vs. improving products and services. Whatever the purpose or benefit is, having commercial and public sector organizations provide a simple statement about why they are using health data would be a good way for them to engage with the public regarding which uses of health data are justifiable from the public’s perspective. Over time, and with deep involvement of members of the public, it may be possible to co-develop other ways to present key information such as icons that convey the information that the public cares the about the most, similar to the “human readable” symbols used for creative commons licences [32], hazardous substances and laundry instructions.

Our proposal to establish plain language for communication with the public is part of a larger ambition of understanding how to involve members of the public in decision making so that we can move beyond informational transparency into participatory transparency and accountability transparency [20,33]. We view this form of communication as an essential step toward having notches on the dial when it comes to public involvement in decisions about health and health-related data. At the low end of the dial, a company or organization using data within their own institution in ways that their clients expect might only need to notify individuals about how data are used and provide information about measures taken to ensure privacy. More communication, engagement and public involvement would be required in cases data are exported from an organization, sold, or linked with data from other organizations, because those practices can increase real and perceived risks to privacy and for uses of data beyond those envisioned when an individual provided their data in the first place. In order to build and maintain public trust when health and health-related data are used without consent, deep and extensive public involvement and engagement will be required to ensure that benefits outweigh risks and that risks are addressed adequately from the public’s perspective.

The health data community is now in a position to initiate and lead a major change in how we communicate with the public as a first step towards broader and deeper public involvement in data-intensive health research and development. We propose to convene a group, including members of the public, and conduct a workshop to refine and expand upon the inputs and ideas presented in this Commentary with the aim of developing model text for plain language communication with the public about uses of consented and non-consented health and health-related data. We encourage interested parties to contact us if they would like to be involved in planning the workshop and developing the materials for it.

## References

[1] McGrail K, Jones K, Akbari A, Bennett T, Boyd A, Carinci F, Cui X, Denaxas S, Dougall N, Ford D, Kirby RS. et al Position Statement on Population Data Science. IJPDS 2018;3(1). 10.23889/ijpds.v3i1.415 PMC8142960 34095517

[2] Carter P, Laurie GT and Dixon-Woods M (2015) The social licence for research: why care.data ran into trouble, Journal of Medical Ethics 2015;41:404-409. 10.1136/medethics-2014-102374 25617016 PMC4431337

[3] Kemp K, Baer Arnold B, Vaile D. My Health Record still isn't safe enough to proceed. It needs more than a band-aid fix. ABC News [Internet] 1 8 2018 [cited 2019 Jan 29] ; Available from: https://www.abc.net.au/news/2018-08-02/my-health-record-still-not-safe/10063026

[4] Smee B. Facebook's data changes will hamper research and oversight, academics warn. The Guardian [Internet] 25 4 2018 [cited 2019 May 5]; Available from: https://www.theguardian.com/technology/2018/apr/25/facebooks-data-changes-will-hamper-research-and-oversight-academics-warn

[5] Andrew-Gee E., Grant T. In the dark: The cost of Canada’s data deficit. The Globe and Mail [Internet] 26 1 2019 [cited 2019 Jan 29]; Available from: https://www.theglobeandmail.com/canada/article-in-the-dark-the-cost-of-canadas-data-deficit/

[6] Osman H. Better access to health data could save $3bn and improve Australians’ health. Healthcare IT News [Internet] 8 1 2019 [cited 2019 Jan 29] Available from: https://www.healthcareit.com.au/article/better-access-health-data-could-save-3bn-and-improve-australians%E2%80%99-health

[7] Boote J, Wong R, Booth A. ‘Talking the talk or walking the walk?’ A bibliometric review of the literature on public involvement in health research published between 1995 and 2009. Health Expectations. 2 2015;18(1):44-57. 10.1111/hex.12007 PMC5060762 23033933

[8] Ministry of Health. [Internet]. Minister's Patient and Family Advisory Council. Government of Ontario, Ministry of Health and Long-Term Care; [cited 2019 May 5]. Available from: http://www.health.gov.on.ca/en/public/programs/pfac/default.aspx/

[9] Smith G. Institutionalizing deliberative mini-publics in Madrid City and German Speaking Belgium – the first steps [Internet]. ConstitutionNet; 2019 [cited 2019 May 5]. Available from: http://constitutionnet.org/news/institutionalizing-deliberative-mini-publics-madrid-city-and-german-speaking-belgium-first

[10] Farrell DM, Suiter J, Harris C. ‘Systematizing’constitutional deliberation: the 2016–18 citizens’ assembly in Ireland. Irish Political Studies. 2 1 2019;34(1):113-123. 10.1080/07907184.2018.1534832

[11] 2018 reform of EU data protection rules [Internet]. European Commission - European Commission 2019 [cited 2019 May 23]. Available from: https://ec.europa.eu/commission/priorities/justice-and-fundamental-rights/data-protection/2018-reform-eu-data-protection-rules_en

[12] Canada's Digital Charter: Trust in a digital world [Internet]. Government of Canada (Innovation, Science and Economic Development Canada); 2019 [cited 2019May23]. Available from: https://www.ic.gc.ca/eic/site/062.nsf/eng/h_00108.html

[13] Freedom of Information and Protection of Privacy Act 3rd Edition 2014 (British Columbia, Canada) Section 35 [cited 2019 May 5] Available from: http://www.bclaws.ca/Recon/document/ID/freeside/96165_00

[14] Personal Health Information Protection Act 2004 (Ontario, Canada) Sections 39, 44 and 45 [cited 2019 May 5] Available from: https://www.ontario.ca/laws/statute/04p03

[15] Regulation GD. Regulation (EU) 2016/679 of the European Parliament and of the Council of 27 April 2016 on the protection of natural persons with regard to the processing of personal data and on the free movement of such data, and repealing Directive 95/46. Official Journal of the European Union (OJ). 2016;59(1-88):294. Available from: https://eur-lex.europa.eu/legal-content/EN/TXT/?qid=1528874672298&uri=CELEX%3A32016R0679

[16] Chassang G. The impact of the EU general data protection regulation on scientific research. ecancermedicalscience. 2017;11 10.3332/ecancer.2017.709 PMC5243137 28144283

[17] Tu JV, Willison DJ, Silver FL, Fang J, Richards JA, Laupacis A, Kapral MK. Impracticability of informed consent in the Registry of the Canadian Stroke Network. New England Journal of Medicine. 1 4 2004;350(14):1414-1421. https://www.nejm.org/doi/full/10.1056/NEJMsa031697 10.1056/NEJMsa031697 15070791

[18] Kho ME, Duffett M, Willison DJ, Cook DJ, Brouwers MC. Written informed consent and selection bias in observational studies using medical records: systematic review. Bmj. 12 3 2009;338:b866 10.1136/bmj.b866 PMC2769263 19282440

[19] Laurie G, Sethi N. Towards Principles–Based Approaches to Governance of Health–Related Research Using Personal Data. European Journal of Risk Regulation. 3 2013;4(1):43-57. 10.1017/S1867299X00002786 24416087 PMC3885861

[20] Aitken M, Tully MP, Porteous C, Denegri S, Cunningham-Burley S, Banner N, Black C, Burgess M, Cross L, van Delden J, Ford E. Consensus statement on public involvement and engagement with data-intensive health research. International Journal of Population Data Science. 2019 Feb 12;4(1). 10.23889/ijpds.v4i1.586 PMC8142968 34095528

[21] Aitken M, Jorre JdS, Pagliari C, Jepson R, Cunningham-Burley S. Public responses to the sharing and linkage of health data for research purposes: a systematic review and thematic synthesis of qualitative studies. BMC medical ethics 2016;17(1):73. 10.1186/s12910-016-0153-x PMC5103425 27832780

[22] Paprica PA, Nunes De Melo M, Schull MJ. Social licence and the general public’s attitudes toward research based on linked administrative health data, a qualitative study, CMAJ 2019 10.9778/cmajo.20180099 PMC6375226 30718354

[23] Teng J, Bentley C, Burgess MM, O'Doherty KC, McGrail KM. Sharing linked data sets for research: results from a deliberative public engagement event in British Columbia, Canada. International Journal of Population Data Science. IJPDS 2019;4(1) 10.23889/ijpds.v4i1.1103. PMC8142623 34095532

[24] The one-way mirror: public attitudes to commercial access to health data. Wellcome Trust. (Ipsos MORI, London (UK)) [Internet] 2016 Mar [cited 2019 May 1] Available from: https://www.ipsos.com/sites/default/files/publication/5200-03/sri-wellcome-trust-commercial-access-to-health-data.pdf

[25] Desai T, Ritchie F, Welpton R. Five Safes: designing data access for research. Deposited 2016 Feb 2, last modified 2017 Jan 30 [cited 2019 Jan 29] Available from: http://eprints.uwe.ac.uk/28124

[26] Lowthian P, Ritchie F. Ensuring the confidentiality of statistical outputs from the ADRN. Deposited 2017 Jun 8, last modified 2017 Aug 17 [cited 2019 Jan 29] Available from: http://eprints.uwe.ac.uk/31986

[27] Parker T. The DataLab of the Australian Bureau of Statistics. Australian Economic Review. 12 2017;50(4):478-483. 10.1111/1467-8462.12246

[28] Smith M. ABOUT THE PHRN. 1 8 2018 [cited 2019 Jan 29] Available from: https://www.pmc.gov.au/sites/default/files/public-submissions/data-sharing-2018/20667.pdf

[29] Google Privacy Policy [Internet] 22 1 2019 [cited 2019 May 1] Available from: https://www.gstatic.com/policies/privacy/pdf/20190122/f3294e95/google_privacy_policy_en.pdf

[30] Clement A, Obar JA. Keeping internet users in the know or in the dark: An analysis of the data privacy transparency of Canadian internet carriers. Journal of Information Policy. 1 6 2016;6(1):294-331. 10.5325/jinfopoli.6.2016.0294

[31] Obar JA, Oeldorf-Hirsch A. The biggest lie on the internet: Ignoring the privacy policies and terms of service policies of social networking services. Information, Communication & Society. 3 7 2018:-1-20. 10.1080/1369118X.2018.1486870

[32] About The Licenses [Internet]. Creative Commons [cited 2019 Jan 29] Available from: https://creativecommons.org/licenses/

[33] Aitken M, Cunningham-Burley S, Pagliari C. Moving from trust to trustworthiness: experiences of public engagement in the Scottish Health Informatics Programme. Sci Public Policy. 2016;43:713. 10.23889/ijpds.v4i1.586 PMC5210028 28066123

